# Identification of a novel survival predictor, CSF2RB, for female lung cancer in never smokers (LCNS) by a bioinformatics analysis

**DOI:** 10.1097/MD.0000000000034019

**Published:** 2023-06-09

**Authors:** Yuan-Yuan Zhou, Xiao-Jun Sun, Jun-Quan Liu, Ling-Li Xiang

**Affiliations:** a KingMed Center for Clinical Laboratory Co., Ltd, Hangzhou, Zhejiang Province, China; b Taizhou Traditional Chinese Medicine Hospital, Taizhou, Jiangsu Province, China.

**Keywords:** bioinformatics analysis, differentially expressed genes (DEGs), female lung cancer in never smokers (LCNS), survival predictor, survival probability

## Abstract

Lung cancer in never smokers (LCNS) has been considered as a separate disease and the 7^th^ cause of cancer-related death worldwide. However, limited research has focused on “female” cohorts, which have presented a higher incidence rate. In this study, the microarray data of lung cancer tissues derived from 54 female lung cancer patients, consisting of 43 nonsmokers and 11 smokers, were selected from GSE2109 dataset. A total of 249 differentially expressed genes (DEGs) including 102 up- and 147 down-regulated genes were identified and further analyzed for gene ontology (GO) terms and Kyoto encyclopedia of genes and genomes (KEGG) pathway enrichment. By constructing protein–protein interaction (PPI) network and calculating key modules, 10 hub genes were screened out. The module analysis of the PPI network presented that the progression of female LCNS was significantly associated with immune response as chemokine activity and lipopolysaccharide response, and these biological processes (BP) might be mediated by chemokine signaling pathway and cytokine-cytokine receptor interaction. Then, survival analysis by Kaplan–Meier (K–M) Plotter online platform presented down-regulated gene colony stimulating factor 2 receptor beta common subunit (CSF2RB) of female LCNS might be involved in poor clinical outcome. Female LCNS with high expression of CSF2RB might be relevant with relative risk reduction of mortality, longer median survival time and higher 5-year survival rate, while female LCNS with low expression of CSF2RB might be implicated in a poor clinical outcome. In short, our results support CSF2RB to be a candidate survival predictor for female LCNS.

## 1. Introduction

Lung cancer, the 5-year survival rate of which used to be <18% for late diagnosis and clinical intervention, is well-known as the leading cause of cancer mortality in both genders worldwide.^[1]^ As low-dose computed tomography (LDCT) being recommended as regular health screening, regardless of age, gender and smoking history, traditional “low-risk” population with the characteristics of young, female and nonsmokers have shown the increasing trend of lung cancer.^[2]^ Though cigarette smoking is the most recognized risk factor of lung cancer, approximately 25% of lung cancer occurs in nonsmokers with a higher prevalence of adenocarcinoma as well as higher incidence in females than in males, especially Asian.^[3–5]^ This leads to lung cancer in never smokers (LCNS) being considered as a separate disease and the 7^th^ cause of cancer-related death all over the world.^[6]^

Furthermore, different carcinogenic pathway, clinicopathological characteristics, epidemiology and natural history were observed among LCNS comparing with ever-smoker patients.^[7,8]^ In a retrospective research covering 718 never smokers and 2514 smokers, nonsmokers were detected with higher incidence rates of well or moderate differentiated tumors and improved 5-year overall survival in pathological T stage IA compared with smokers.^[4]^ Many recent studies focused on the identification of risk factors except for tobacco smoking and found other environmental risk factors including exposure to secondhand smoke, air pollution, kitchen fume, radon, asbestos, hormones, infection of oncogenic viruses, and inflammatory diseases.^[2,7,9]^ Nevertheless, genetic predisposition performs more predominantly for LCNS.^[6,8]^ Though researchers have realized that LCNS might be a different disease from traditionally studied cases attributed to tobacco smoking, the molecular mechanism in tumorigenesis of LCNS still remains unclarified.

New technologies such as genomic sequencing and microarray chips and profiling cancer-specific gene expression programs provide groundbreaking ways to elucidate fundamental cellular processes and disease etiology via high-throughput experimental platforms. Bioinformatics tools serve as an important bridge connecting basic biological sciences to clinical diagnostics, therapeutics, prediction of disease progression and clinical outcome.^[10]^ Previous studies have focused on LCNS without gender classification or have been limited to paired tumor and adjacent normal lung tissue specimens.^[11–14]^ Therefore, in order to identify candidate hub genes and further elucidate the complex biological mechanisms involved in female LCNS, a bioinformatics analysis based on high-throughput gene expression profiles and unbiased bioinformatics tools is warranted.

In the present study, microarray data of female lung cancer patients were screened from the Gene Expression Omnibus (GEO, http://www.ncbi.nlm.nih.gov/geo) database and divided into a smokers group and a nonsmokers group. Differentially expressed genes (DEGs) were identified and further analyzed functional enrichment and protein–protein interaction (PPI) network. Then hub genes were calculated and analyzed in significant modules, following by Kaplan–Meier (K–M) survival analysis. The selected candidate gene colony stimulating factor 2 receptor beta common subunit (CSF2RB) could be a potential survival predictor for female LCNS, which could also provide insight into the molecular mechanism of disease subtype and develop target genes for diagnosis, prognosis and therapeutics.

## 2. Results

### 2.1. Data preprocessing

The microarray data of lung cancer tissue samples from 54 female lung cancer patients, including 43 nonsmokers and 11 smokers, were selected from 2158 different tumor tissue samples in the GSE2109, which contained gene expression profiling of 54,675 common genes. The characteristics of patients are detailed in Table 1.

**Table 1:** The characteristics of patients in this study.

Sample title	Geo accession	Sample source	Patient age	Gender	Ethnic background	Tobacco use	Yr of tobacco use	Alcohol consumption	Family history of cancer
Lung - 202033	GSM138009	Lung	40–50	Female	American Indian	No	0	No	Yes
Lung - 187212	GSM117757	Lung	40–50	Female	Caucasian	No	0	No	Yes
Lung - 254375	GSM152767	Lung	40–50	Female	Caucasian	No	0	No	Yes
Lung - 251053	GSM152755	Lung	50–60	Female	Caucasian	No	0	No	Yes
Lung - 8258	GSM38104	Lung	70–80	Female	Caucasian	No	0	No	Yes
Lung - 22235	GSM46860	Lung	70–80	Female	Caucasian	No	0	No	Yes
Lung - 362593	GSM231891	Lung	70–80	Female	Caucasian	No	0	No	Yes
Lung - 151625	GSM152592	Lung	80–90	Female	Caucasian	No	0	No	Yes
Lung - 371434	GSM203787	Lung	80–90	Female	Caucasian	No	0	No	Yes
Lung - 465774	GSM353912	Lung	80–89	Female	Caucasian	No	0	Yes	No
Lung - 36311	GSM76572	Lung	50–60	Female	Caucasian	No	0	No	No
Lung - 181030	GSM137931	Lung	60–70	Female	Caucasian	Yes	11–15	No	Yes
Lung - 4446	GSM117772	Lung	70–80	Female	Caucasian	Yes	11–15	No	Yes
Lung - 22255	GSM46868	Lung	60–70	Female	Caucasian	Yes	16–20	No	Yes
Lung - 170806	GSM137916	Lung	60–70	Female	Caucasian	Yes	16–20	No	Yes
Lung - 465762	GSM353908	Lung	80–89	Female	Caucasian	Yes	16–20	No	Yes
Lung - 2491	GSM46909	Lung	None	Female	None	Yes	16–20	No	Yes
Lung - 199377	GSM138001	Lung	70–80	Female	Caucasian	Yes	26–30	No	Yes
Lung - 20283	GSM88983	Lung	60–70	Female	Caucasian	Yes	31–35	No	Yes
Lung - 465775	GSM353913	Lung	60–69	Female	Caucasian	Yes	36–40	No	Yes
Lung - 36318	GSM76574	Lung	50–60	Female	Caucasian	Yes	41–45	No	Yes
Lung - 21802	GSM117770	Lung	60–70	Female	Caucasian	Yes	41–45	No	Yes
Lung - 101192	GSM102455	Lung	70–80	Female	Caucasian	Yes	41–45	No	Yes
Lung - 405008	GSM277695	Lung	70–80	Female	Caucasian	Yes	41–45	No	Yes
Lung - 174063	GSM117714	Lung	60–70	Female	Caucasian	Yes	46–50	No	Yes
Lung - 362608	GSM231896	Lung	60–70	Female	Caucasian	Yes	46–50	No	Yes
Lung - 5225	GSM53170	Lung	70–80	Female	Caucasian	Yes	56–60	No	Yes
Lung - 31907	GSM46961	Lung	80–90	Female	Caucasian	Yes	66–70	No	Yes
Lung - 8284	GSM46975	Lung	50–60	Female	Caucasian	Yes	11–15	Yes	Yes
Lung - 205700	GSM203643	Lung	60–70	Female	Caucasian	Yes	11–15	Yes	Yes
Lung - 101183	GSM102450	Lung	40–50	Female	Caucasian	Yes	16–20	Yes	Yes
Lung - 6991	GSM76630	Lung	70–80	Female	Caucasian	Yes	16–20	Yes	Yes
Lung - 31579	GSM88997	Lung	50–60	Female	Caucasian	Yes	36–40	Yes	Yes
Lung - 101138	GSM88959	Lung	60–70	Female	Caucasian	Yes	36–40	Yes	Yes
Lung - 22208	GSM46850	Lung	70–80	Female	Caucasian	Yes	36–40	Yes	Yes
Lung - 31812	GSM46936	Lung	80–90	Female	Caucasian	Yes	46–50	Yes	Yes
Lung - 433111	GSM301680	Lung	60–69	Female	Caucasian	Yes	51–55	Yes	Yes
Lung - 20272	GSM88981	Lung	80–90	Female	Caucasian	Yes	16–20	None	Yes
Lung - 101178	GSM102447	Lung	70–80	Female	Asian	Yes	26–30	No	No
Lung - 161511	GSM117689	Lung	60–70	Female	African-American	Yes	36–40	No	No
Lung - 182730	GSM117731	Lung	60–70	Female	Caucasian	Yes	31–35	Yes	No
Lung - 154213	GSM137910	Lung	60–70	Female	Caucasian	Yes	21–25	No	No
Lung - 219599	GSM152681	Lung	50–60	Female	Caucasian	Yes	46–50	Yes	No
Lung - 242924	GSM179827	Lung	60–70	Female	Caucasian	Yes	26–30	Yes	No
Lung - 346001	GSM231885	Lung	60–70	Female	Caucasian	Yes	46–50	No	No
Lung - 346006	GSM277678	Lung	70–80	Female	Caucasian	Yes	26–30	No	No
Lung - 416790	GSM277696	Lung	70–80	Female	Caucasian	Yes	31–35	No	No
Lung - 31822	GSM46941	Lung	60–70	Female	Caucasian	Yes	41–45	No	No
Lung - 31903	GSM46960	Lung	50–60	Female	Caucasian	Yes	36–40	Yes	No
Lung - 20275	GSM76488	Lung	60–70	Female	Caucasian	Yes	36–40	No	No
Lung - 4455	GSM76587	Lung	70–80	Female	Caucasian	Yes	36–40	Yes	No
Lung - 45125	GSM76590	Lung	60–70	Female	Caucasian	Yes	26–30	Yes	No
Lung - 5132	GSM76595	Lung	60–70	Female	Caucasian	Yes	31–35	Yes	No
Lung - 8067	GSM76647	Lung	60–70	Female	Caucasian	Yes	36–40	No	No

GEO = gene expression omnibus.

### 2.2. DEGs screening

**Figure 1.**
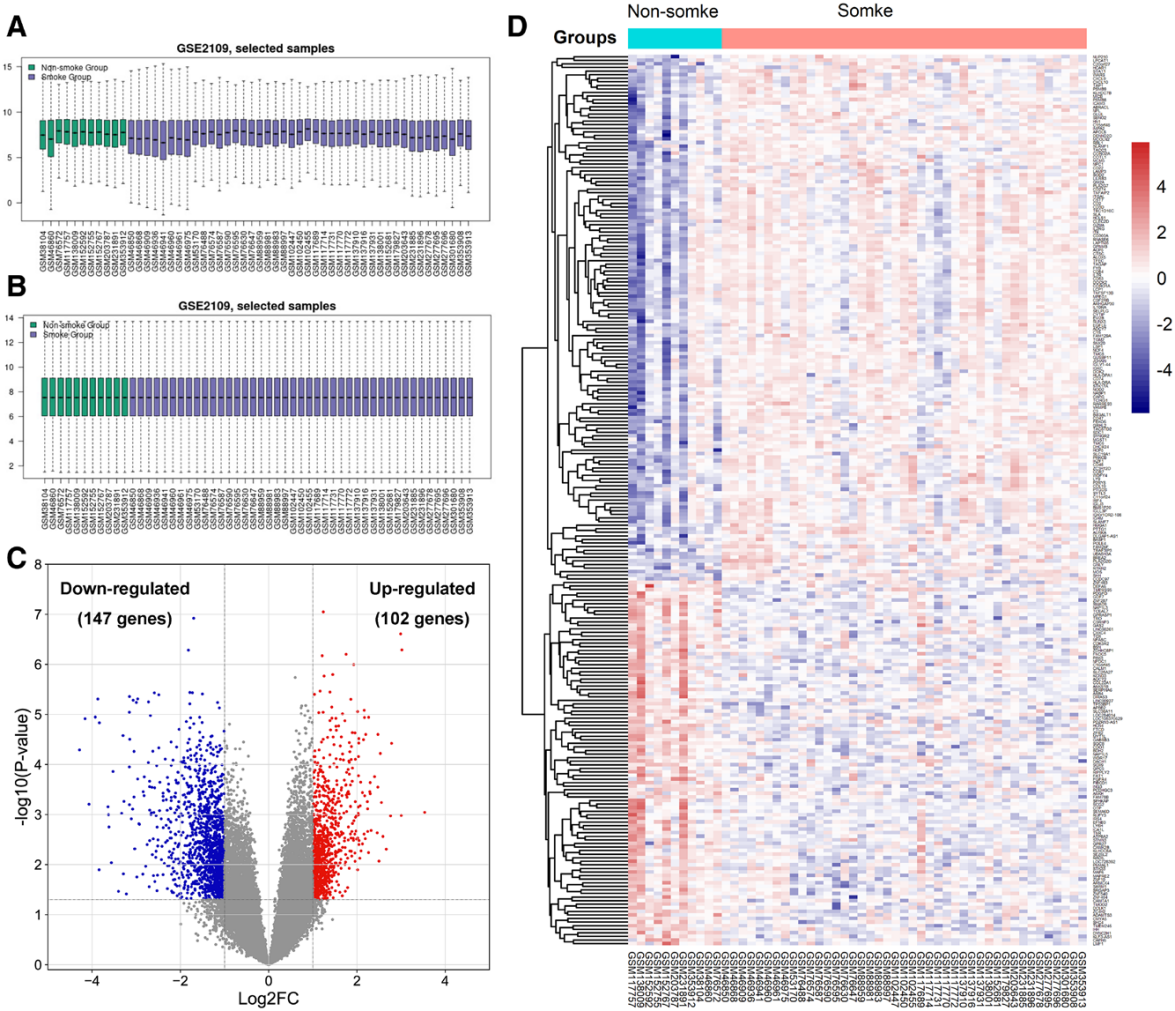
Normalization of gene expression data, volcano plot and heatmap of DEGs in mRNA expression profiling datasets. (A and B) Gene expression profiles (A) prior to normalization and (B) after normalization. (C) Volcano plot of DEGs between female LCNS group and female lung cancer in smokers group. (D) Heat map of DEGs expression in the dataset GSE2109. DEGs = differentially expressed genes, FC = fold change, LCNS = lung cancer in never smokers.

The raw data of GSE2109 were normalized as presented in Figure 1A and B. A total of 379 significant DEGs in female LCNS compared with female lung cancer in smokers were screened. After removing invalid probes without unique corresponding gene symbols, 249 remaining DEGs were identified, consisting of 102 up-regulated genes and 147 down-regulated genes. The DEGs distribution was visualized by a volcano plot in Figure 1C. In addition, the differential expression analysis was presented in a heatmap in Figure 1D.

### 2.3. Gene ontology (GO) terms and Kyoto encyclopedia of genes and genomes (KEGG) pathway enrichment analysis of DEGs

**Figure 2.**
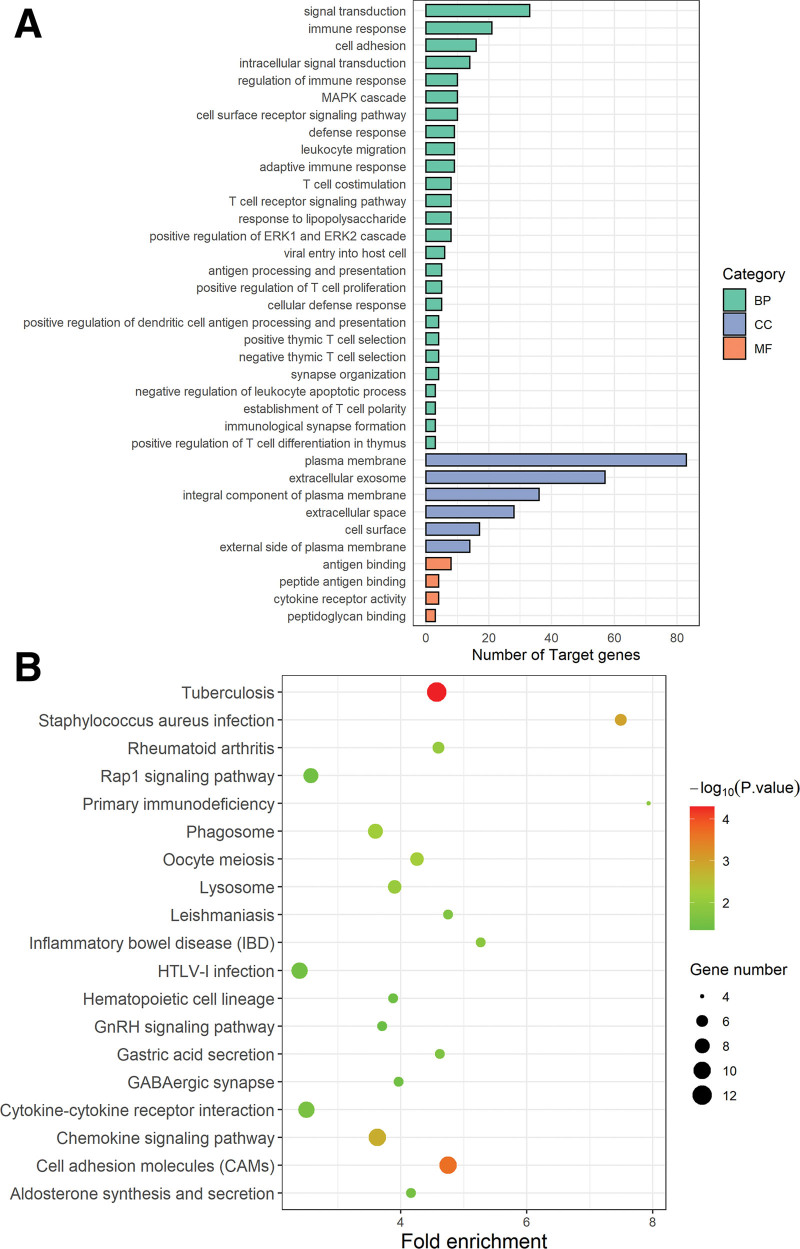
GO and KEGG analysis of DEGs. (A) The significant GO categories of biological process (BP), cellular component (CC) and molecular function (MF) for DEGs. *P*<.01 was considered significantly. (B) The significant KEGG pathway analysis for DEGs. *P*<.05 was considered significantly. DEGs, differentially expressed genes, GO = gene ontology, KEGG = Kyoto encyclopedia of genes and genomes.

The biological function of DEGs were studied by GO enrichment analysis, including biological process (BP), cellular component and molecular function for DEGs. As shown in Figure 2A, the significant GO categories in BP were enriched in signal transduction (GO:0007165), immune response (GO:0006955), cell adhesion (GO:0007155), intracellular signal transduction (GO:0035556), regulation of immune response (GO:0050776), MAPK cascade (GO:0000165), and cell surface receptor signaling pathway (GO:0007166) with a *P* value <.01 and gene number more than 10. The significant GO categories in cellular component were enriched in membrane and related integral component, such as plasma membrane (GO:0005886), extracellular exosome (GO:0070062), integral component of plasma membrane (GO:0005887), extracellular space (GO:0005615), cell surface (GO:0009986) and external side of plasma membrane (GO:0009897). The significant GO categories in molecular function were enriched in (peptide) antigen binding (GO:0042605 and GO:0003823) and cytokine receptor activity (GO:0004896) with a *P* value <.01 and gene number more than 4. KEGG pathway enrichment analysis shown that the DEGs were significantly enriched in Tuberculosis (hsa05152), Cell adhesion molecules (CAMs) (hsa04514), Chemokine signaling pathway (hsa04062), Phagosome (hsa04145), Cytokine-cytokine receptor interaction (hsa04060), HTLV-I infection (hsa05166) and Rap1 signaling pathway (hsa04015) with a *P* value <.05 and gene number more than 8 (Fig. 2B).

### 2.4. PPI network construction and module analysis

**Figure 3.**
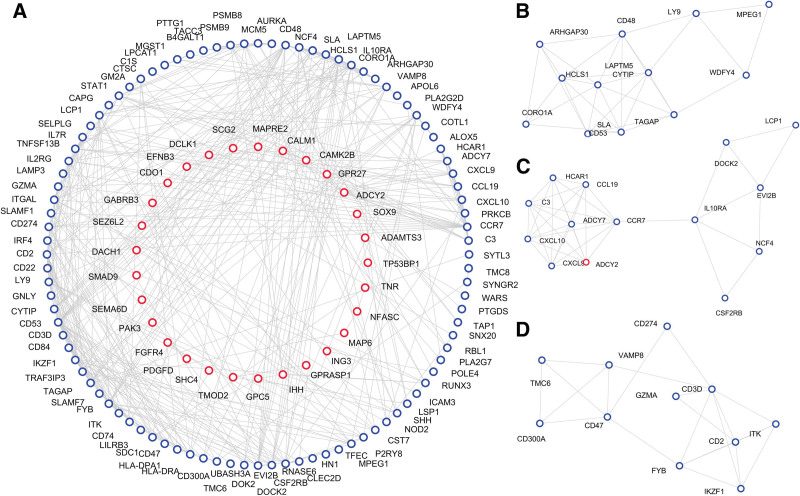
PPI network of DEGs and modules analysis. (A) The PPI network of DEGs. The interaction score 0.500 was set as the threshold. (B–D) The top 3 modules of PPI network. The red circles represent the nodes of up-regulated DEGs. The blue circles represent the nodes of down-regulated DEGs. DEGs = differentially expressed genes, PPI = protein–protein interaction.

The PPI network was constructed by online Search Tool for the Retrieval of Interacting Genes/Proteins database with the threshold of the interaction score 0.500 (above the median confidence). Nodes without connections were removed and then the PPI network consisted of 218 nodes and 406 edges with average local clustering coefficient 0.303 (PPI enrichment *P* value<1.0^–16^) was visualized via Cytoscape (Fig. 3A). Subsequently, hub genes were ranked by Degree and top 10 nodes were screened via cytoHubba plug-in (Table 2).

**Table 2:** Top 10 hub genes in PPI network ranked by degree method.

Rank	Name	Score
1	CD53	25
2	CD2	22
3	FYB	21
3	CCR7	21
5	IL10RA	20
5	CD48	19
5	LAPTM5	19
8	IKZF1	18
9	CSF2RB	15
10	ITGAL	15

CSF2RB = colony stimulating factor 2 receptor beta common subunit, PPI = protein–protein interaction.

The significant 3 modules were calculated with molecular complex detection plug-in (Fig. 3B–D) in order to further analyze their GO and KEGG functional clusters based on modules with BiNGO plug-in and database for annotation, visualization and integrated discovery (DAVID) database. Only GO terms and KEGG pathways were found significant in the second top module (Table 3, *P* < .05). The KEGG analysis of module genes shown that the top 3 modules (i.e., the second one) were mainly associated with Chemokine signaling pathway (hsa04062) and Cytokine-cytokine receptor interaction (hsa04060). Besides, the GO analyses indicated that these module genes were enriched in chemokine activity (GO:0008009), response to lipopolysaccharide (GO:0032496) and other immune response related BP.

**Table 3:** The GO and KEGG enrichment analysis of the genes in the top 3 modules (*P*<.05).

Category	Term	Genes	*P* value	FDR
GOTERM_BP	GO:0032496~response to lipopolysaccharide	CXCL10, CXCL9, IL10RA, CCR7, CSF2RB	4.08E-06	3.37E-04
GOTERM_BP	GO:0006955~immune response	C3, CXCL10, CXCL9, NCF4, CCR7, CCL19	6.63E-06	3.37E-04
GOTERM_BP	GO:0001768~establishment of T cell polarity	CCR7, CCL19, DOCK2	7.01E-06	3.37E-04
GOTERM_BP	GO:0006935~chemotaxis	CXCL10, CXCL9, CCR7, DOCK2	7.85E-05	2.82E-03
GOTERM_BP	GO:0006954~inflammatory response	C3, CXCL10, CXCL9, CCR7, CCL19	1.10E-04	3.15E-03
GOTERM_BP	GO:0007186~G-protein coupled receptor signaling pathway	C3, CXCL10, CXCL9, HCAR1, CCR7, CCL19	2.51E-04	6.03E-03
GOTERM_BP	GO:0060326~cell chemotaxis	CXCL10, CXCL9, CCL19	9.50E-04	1.95E-02
GOTERM_BP	GO:0070098~chemokine-mediated signaling pathway	CXCL10, CXCL9, CCL19	1.13E-03	2.04E-02
GOTERM_BP	GO:0072610~interleukin-12 secretion	CCR7, CCL19	1.43E-03	2.29E-02
GOTERM_BP	GO:0002408~myeloid dendritic cell chemotaxis	CCR7, CCL19	2.14E-03	2.80E-02
GOTERM_BP	GO:0071731~response to nitric oxide	CCR7, CCL19	2.14E-03	2.80E-02
GOTERM_BP	GO:0097029~mature conventional dendritic cell differentiation	CCR7, CCL19	3.57E-03	4.28E-02
GOTERM_BP	GO:2000107~negative regulation of leukocyte apoptotic process	CCR7, CCL19	4.28E-03	4.40E-02
GOTERM_BP	GO:0030816~positive regulation of cAMP metabolic process	CXCL10, CXCL9	4.28E-03	4.40E-02
GOTERM_BP	GO:0002606~positive regulation of dendritic cell antigen processing and presentation	CCR7, CCL19	4.99E-03	4.79E-02
GOTERM_MF	GO:0008009~chemokine activity	CXCL10, CXCL9, CCL19	5.35E-04	2.73E-02
KEGG_PATHWAY	hsa04062:Chemokine signaling pathway	CXCL10, CXCL9, CCR7, ADCY2, CCL19, DOCK2, ADCY7	1.49E-07	8.93E-06
KEGG_PATHWAY	hsa04060:Cytokine-cytokine receptor interaction	CXCL10, CXCL9, IL10RA, CCR7, CSF2RB, CCL19	2.05E-05	6.15E-04

BP = biological process, CSF2RB = colony stimulating factor 2 receptor beta common subunit, FDR = false positive rate, GO = gene ontology, KEGG = Kyoto encyclopedia of genes and genomes, MF = molecular function.

### 2.5. K–M survival analysis and candidate genes selection

Ten screened hub genes from PPI network were further studied with K–M Plotter online platform. The overall survival analyses were detailed in Table 4, which unraveled significant difference between low and high expression groups of CSF2RB gene in female LCNS (*P* < .05). On the other hand, no statistically significant difference was found with diverse CSF2RB gene expression levels for female lung cancer in smokers (*P* > .05).

**Table 4:** The overall survival analyses of the hub genes.

Gene symbol	Female lung cancer in smokers				Female lung cancer in never smokers (LCNS)			
HR	Log rank *P*	Low expression cohort (mo)	High expression cohort (mo)	HR	Log rank *P*	Low expression cohort (mo)	High expression cohort (mo)
CD53	0.98 (0.7–1.38)	.9101	95.5	95	0.84 (0.45–1.58)	.5858	69	75.73
CD2	0.74 (0.52–1.03)	.0759	91	119.5	1 (0.53–1.88)	.9975	75.43	72
FYB	0.86 (0.38–1.96)	.7263	40.21	68	0.89 (0.36–2.18)	.7913	72	88.7
CCR7	0.87 (0.62–1.22)	.4053	96.1	85.65	1.54 (0.8–2.97)	.1972	NA	NA
IL10RA	0.69 (0.49–0.98)	.0348	71	96.1	0.59 (0.31–1.12)	.1026	54.2	75.73
CD48	1.03 (0.73–1.46)	.8518	95.5	85.65	1.6 (0.84–3.05)	.1522	75.43	69
LAPTM5	0.86 (0.61–1.21)	.3875	95.5	95	1.26 (0.67–2.4)	.473	96	69
IKZF1	1.59 (0.69–3.68)	.2719	91	47.63	0.76 (0.31–1.89)	.5554	75.43	88.7
CSF2RB	0.89 (0.63–1.25)	.501	95	95.5	0.36 (0.18–0.7)	.0019	49	88.7
ITGAL	0.65 (0.46–0.91)	.0123	62.47	95	1.42 (0.75–2.7)	.2846	75.73	69

CSF2RB = colony stimulating factor 2 receptor beta common subunit.

**Figure 4.**
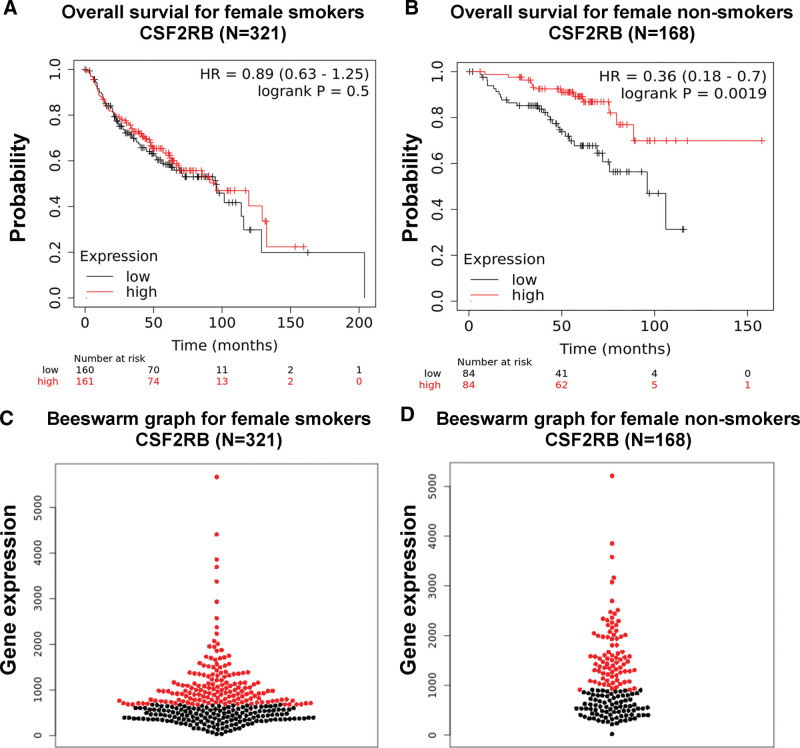
Overall survival analyses of candidate gene CSF2RB. (A) The overall survival for 321 female smokers with lung cancer. (B) The overall survival for 168 female nonsmokers with lung cancer. (C) The beeswarm graph for 321 female smokers with lung cancer. (D) The beeswarm graph for 168 female nonsmokers with lung cancer. The red lines and dots represent the patients with high expression of CSF2RB. The black lines and dots represent the patients with low expression of CSF2RB. Groups were split by median expression levels. CSF2RB = colony stimulating factor 2 receptor beta common subunit.

Among 168 “only those never smoked” female lung cancer patients, 84 patients with high expression of CSF2RB (Fig. 4A, log-rank *P* = .0019; hazard ratios [HR] = 0.36; 95% confidence intervals [CI]: 0.18–0.7) resulted in a significantly decreased risk of mortality compared with 84 patients with low expression of CSF2RB gene. However, in 321 “exclude those never smoked” female lung cancer patients, no significant difference was observed between 161 patients with high expression and 160 patients with low expression of CSF2RB (Fig. 4B, log-rank *P* = .501; HR = 0.89; 95% CI: 0.63–1.25). Overall, female lung cancer patients with low expression of CSF2RB were significantly correlated with a poor prognosis. Patients with different gene expression were split by median expression levels and grouping information were illustrated in beeswarm graphs (Fig. 4C and D).

### 2.6. Low expression level of CSF2RB in lung cancer tissues is associated with poor clinical outcome

In the K–M survival curves (Fig. 4A and B), the vertical axis represents survival probability, while the horizontal axis represents survival time (months). Based on clinical survival data (Table 4), the median survival time for CSF2RB-low-expression patients was remarkably lower than that for CSF2RB-high-expression patients in 168 female LCNS (49 months vs 88.7 months, *P* = .0019), while among 321 female patients with smoking history, the median survival time for patients of CSF2RB low-expression and high-expression shown no significant difference (95 months vs 95.5 months, *P* = .501).

Similarly, the rate of 5-year survival in female LCNS group with high expression of CSF2RB (86%) was significantly higher than that with low expression of CSF2RB (67%, *P* = .0019). In contrast, the 5-years survival probability presented no obvious difference in smoking patients with low gene expression of CSF2RB compared to that with high expression (58% vs 62%, *P* = .501).

## 3. Discussion

In the current study, the microarray data of lung cancer tissues from 54 female lung cancer patients, consisting of 43 nonsmokers and 11 smokers, were selected from GSE2109 dataset. A total of 249 valid DEGs, including 102 up-regulated and 147 down-regulated genes, were identified and further analyzed for GO terms and KEGG pathway enrichment. After the construction of PPI network for these DEGs, top 10 hub genes and 3 significant modules were calculated, following by further analyzing their GO and KEGG functional cluster. The KEGG pathway enrichment was mainly associated with chemokine signaling pathway and cytokine-cytokine receptor interaction, while the GO terms were enriched in chemokine activity, response to lipopolysaccharide and other immune response related BP. Moreover, the overall survival analyses of 10 screened hub genes within 168 “only those never smoked” patients revealed a significant difference between low and high expression groups of CSF2RB gene in female LCNS. Instead, in 321 “exclude those never smoked” patients, no such statistically significant difference was found among female lung cancer in smokers. In conclusion, female LCNS with high expression of CSF2RB were observed relative risk reduction of mortality, longer median survival time and higher 5-year survival rate, while female LCNS with low expression of CSF2RB were significantly correlated with a poor prognosis.

Human CSF2RB gene, also known as CD131, CDw131, IL3RB, IL5RB, SMDP5, betaGMR, is mapped to chromosome 22q12.3 and could express in most cells. The protein encoded by this gene is the common beta chain of the high affinity receptor for GM-CSF, IL-3, and IL-5 cytokines. Defects in this gene have been reported to be associated with pulmonary alveolar proteinosis,^[15,16]^ Crohn disease (CD),^[17]^ Down Syndrome,^[18]^ while higher expression of gene CSF2RB was associated with better disease-free survival (DFS) for stage II non-small cell lung carcinoma patients with curative resection.^[19]^ Previous reports had elucidated that GM-CSF and its receptor might be key players in the steady-state functional regulation of lung physiology.^[15]^ Besides, CSF2RB was identified as important in the tumor microenvironment of lung adenocarcinoma (LUAD).^[20]^ In our study, the module analysis of the PPI network presented that the progression of female LCNS was significantly associated with immune response as chemokine activity and lipopolysaccharide response, and these BP might be mediated by chemokine signaling pathway and cytokine-cytokine receptor interaction.

Although recent bioinformatics studies have aimed to investigate hub genes for lung cancer and have reported that CSF2RB might be a survival-related gene of LUAD,^[11,13]^ limited research has been focused on “female” nonsmokers with lung cancer cohorts. As previously reported, 120 samples of paired tumor and adjacent normal lung tissue specimens from the same patient were collected to study non-small cell lung carcinoma in nonsmoking women for the reason that tumor tissues seem highly heterogeneous between diverse individuals.^[14]^ Based on the above research, 98 lung cancer samples of female cancer patients who never smoked and 4 normal female lung tissues from the other dataset were combined straightly to analyze for the same purpose.^[12]^ However, it is still necessary to find DEGs not only between tumor tissues and adjacent normal tissues of the same individuals but also among tumor tissues derived from patients with diverse smoking history, because tumorigenesis is known to relate with genetics and it might be paradox to think the expression and function of vital genes different between tumor and adjacent normal tissues with preconceptions.

In the present study, the down-regulated gene CSF2RB was found associated with female LCNS compared to female lung cancer in smokers and its low expression in tumor tissues might be implicated in poor clinical outcome by a bioinformatics analysis. The above conjecture about the potential of CSF2RB to be a survival-related gene of LUAD is consistent with our results,^[13]^ which might be due to the pathologic differences that non-small-cell lung cancer (mainly adenocarcinoma) happens more common in never and light smokers. This brought us to identify CSF2RB as a candidate survival predictor for female LCNS, which could also be applied as potential diagnostic biomarkers and therapeutic targets. However, the sample size is limited and comprehensively analyzing data from different projects is necessary to enhance clinical reference. Furthermore, as weakly supportive as in other bioinformatics research, in vivo and in vitro functional experiments are required to verify the relationship between CSF2RB gene and female LCNS and to investigate the molecular mechanism.

## 4. Conclusions

In summary, this study identified the relationship between down-regulated gene CSF2RB and female LCNS by bioinformatics analysis. Furthermore, female LCNS with high expression of CSF2RB might be relevant with relative risk reduction of mortality, longer median survival time and higher 5-year survival rate, while female LCNS with low expression of CSF2RB might be implicated in a poor clinical outcome. Although our results supported CSF2RB to be a candidate survival predictor and potential therapeutic target for female LCNS, some limitations were present in the study: this study is a bioinformatics analysis based on an open database; the sample size is limited and comprehensively analyzing data from different projects is necessary for further investigation; further study would be required for experimental verification and molecular mechanism clarification.

## 5. Methods

### 5.1. Microarray data information

The dataset of GSE2109, downloaded from the GEO database, is based on the GPL570 platform (Affymetrix Human Genome U133 Plus 2.0 Array). All lung cancer tissue samples from female lung cancer patients were selected and divided into 2 groups: nonsmokers and smokers. The following research steps were detailed as Flow Diagram in the Supplementary file, http://links.lww.com/MD/J125.

### 5.2. DEGs analysis

The DEGs between female LCNS and female lung cancer in smokers were screened utilizing a public web-tool GEO2R, which could compare groups of Samples in GSE2109 dataset for DEGs, visualize DEGs by graphic plots and assess dataset quality. The comparison and normalization of the microarray data were performed using the GEOquery and limmaR packages from the Bioconductor project with significance level cutoff (adjusted *P* values, adj. *P*) <05 and log_2_|fold change|>1. The Benjamini-Hochberg procedure was employed to diminish the false positive rate by default.

### 5.3. GO terms and KEGG pathway enrichment analysis

In order to analyze the biological classification of DEGs, Gene Ontology (GO) and KEGG pathway enrichment analyses were conducted by DAVID, which is an online biological information database for visualization and integrated the functional annotation information of genes and proteins (https://david.ncifcrf.gov/). *P* < .01 and *P* < .05 were considered to be statistically significant for GO and KEGG analyses, respectively.

### 5.4. PPI network construction and module analysis

The online database Search Tool for the Retrieval of Interacting Genes was utilized to reveal the interaction among DEGs and construct the PPI network with interaction score of 0.500 as the threshold,^[21]^ which was then visualized via Cytoscape software (Version 3.8.2).^[22,23]^ Besides, hub genes were calculated by Cytoscape plug-in cytoHubba with top 10 nodes ranked by Degree, while module analysis was performed by Cytoscape plug-in molecular complex detection with default parameters and further GO function and KEGG pathway enrichment analysis were conducted with Cytoscape plug-in BiNGO and DAVID database. False positive rate <0.05 was thought as significant.

### 5.5. K–M survival analysis and candidate genes selection

Samples from 321 “exclude those never smoked” and 168 “only those never smoked” female lung cancer patients were analyzed separately for 10 screened hub genes via K–M Plotter online platform.^[24]^ Survival analysis were investigated by K–M method with log-rank test, and K–M plots were used to assess the difference between groups of patients with different gene expression, which were split by median expression levels. HR and 95% CI were used to assess the relative risk of overall survival. The screened candidate genes could serve as the potential predictive biomarkers of survival time and therapeutic targets for prolonging the survival of female non-smoked patients with lung cancer.

## Author contributions

**Conceptualization:** Xiao-Jun Sun, Jun-Quan Liu.

**Data curation:** Ling-Li Xiang.

**Formal analysis:** Yuan-Yuan Zhou, Ling-Li Xiang.

**Investigation:** Jun-Quan Liu.

**Project administration:** Yuan-Yuan Zhou.

**Methodology:** Xiao-Jun Sun.

**Resources:** Xiao-Jun Sun.

**Software:** Xiao-Jun Sun.

**Supervision:** Jun-Quan Liu.

**Writing – original draft:** Yuan-Yuan Zhou, Ling-Li Xiang.

**Writing – review & editing:** Yuan-Yuan Zhou, Xiao-Jun Sun, Jun-Quan Liu, Ling-Li Xiang.

## Supplementary Material


