# Supplementary material for: Identification of a novel survival predictor, CSF2RB, for female lung cancer in never smokers (LCNS) by a bioinformatics analysis
Source: Medicine (Baltimore). 2023 Jun 9;102(23):e34019. doi: 10.1097/MD.0000000000034019 (PMC10256432; doi:10.1097/MD.0000000000034019)

Supplementary File :

The dataset of GSE2109, downloaded from the GEO database, is based on the GPL570 platform (Affymetrix Human Genome U133 Plus 2.0 Array). All lung cancer tissue samples from female lung cancer patients were selected and divided into two groups: non-smokers and smokers. The following research steps were detailed as Flow Diagram in the supplementary file.

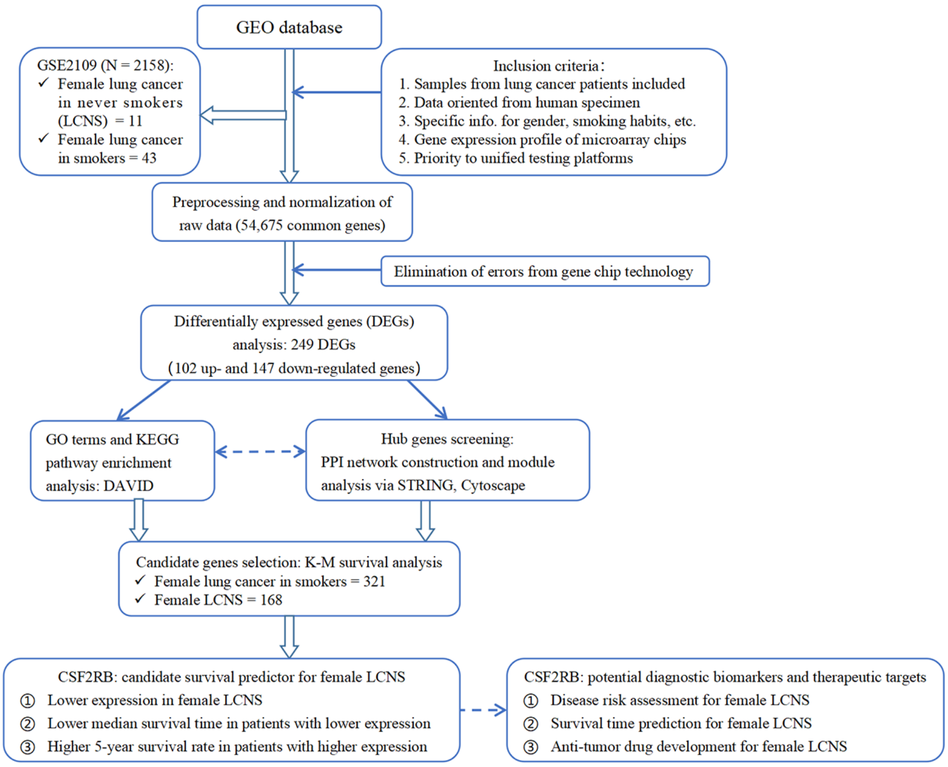

Supplement: Supplementary file 1 [file medi-102-e34019-s001.pdf]
